# Is it possible for parents to endure a stillbirth? Initial experiences, perceptions and strategies: individual in-depth interviews in Sweden 2021–2023

**DOI:** 10.1186/s12884-024-07055-0

**Published:** 2025-01-03

**Authors:** Berit Höglund, Ingegerd Hildingsson

**Affiliations:** 1 Department of Women’s and Children’s Health, Uppsala University, Uppsala, 751 85 Sweden; 2 Department of Nursing, Mid Sweden University, Sundsvall, Sweden; 3 Department of Nursing, Umeå University, Umeå, Sweden

**Keywords:** Coping strategies, Foetal movements, Grieving process, In-depth interviews, Parents, Professionals, Stillbirth

## Abstract

**Background:**

Stillbirth occurs at a rate of 3.0 per thousand in Sweden. However, few studies have focused on the initial experiences of parents facing a stillbirth. The aim of this qualitative study is to deepen and broadly explore parents’ initial experiences, perceptions, internal processes and strategies from the moment of suspicion or awareness of stillbirth until one month after the event.

**Methods:**

Ten individual in-depth interviews were conducted between 2021 and 2023, and data were evaluated using thematic network analysis.

**Results:**

Two key themes emerged: ‘*Following the journey – from suspicion to acceptance’* and ‘*Support, structured activities and processes after stillbirth’.* These themes captured the significant consequences of a sudden, unexpected and devastating end to pregnancy. The suspicion and eventual diagnosis of stillbirth were initially associated with sudden discomfort, fear, overwhelming grief, and intense pain. Nevertheless, a vaginal birth was regarded as the optimal mode of delivery for both physical and emotional wellbeing. Caring for the stillborn baby through physical proximity for an extended period of time helped parents comprehend and cope with their grief, while also affirming their sense of parenthood.

**Conclusions:**

This study sheds light on the profound and devastating impact of stillbirth on parents who are confronted with the loss of their long-awaited and cherished baby. The intense grief and pain experienced by parents during the first month after stillbirth were described as an ongoing heavy burden, persisting day and night, and reflected in poor/very poor mental health. Despite the immense challenges faced by parents, the study highlights the importance of developing individual coping strategies to deal with this tragic and irreversible life-changing event.

**Supplementary Information:**

The online version contains supplementary material available at 10.1186/s12884-024-07055-0.

## Background

Losing a baby through stillbirth profoundly affects both women and men throughout their lifetime [1, 2]. In Sweden, stillbirth is defined as the death of a baby in utero after 22 completed weeks of gestational [3]. In 2022, 315 babies were stillborn after 22 completed weeks of pregnancy in Sweden, with 294 (93%) diagnosed with stillbirth before labour commenced. In 2021, most stillbirths occurred between 32 and 36 completed weeks of gestational [3]. Although the rate of stillbirth has decreased in recent years due to advancements in healthcare practices, this irreversible and life-changing event continues to occur. A recent study identified two primary causes of stillbirth: placental complications (40.5%) and umbilical cord complications (30.2%) [4]. Another study from 2014 confirmed various complications associated with gestational age at the time of stillbirth [5]. The study also highlighted that preterm stillbirths were associated with a higher prevalence of conditions such as placental abruption, preeclampsia or hypertension, malformations or chromosomal abnormalities, and intrauterine growth restriction or placental insufficiency. In contrast, umbilical cord complications, birth hypoxia, and infections were more commonly associated with post-term stillbirths [5].

Identifying the exact cause of stillbirth is important for parents, and it is also critical for obtaining essential information that may help prevent stillbirth in future pregnancies. Even when an autopsy is performed, it can be difficult to determine the cause of each individual stillbirth [6, 7]. Moreover, other studies describe how bereaved parents experience feelings of shame, self-blame, devaluation of motherhood, and discrimination following a stillbirth [1, 2, 8–10]. Australian studies have also reported that parents perceived that healthcare providers did not inform them about the possibility of stillbirth during antenatal care [2, 10, 11].

There are relatively few studies on labouring with an antepartum foetal death. However, a recent study affirms that parents’ experiences of labour after foetal death in utero provide important perspectives for intrapartum care [12]. Furthermore, that study describes how parents want healthcare providers to facilitate their choices, maintain their sense of control, and respect their autonomy and agency. In addition, parents wish to feel that they received the best possible care during stillbirth. Another study emphasises the profound and pervasive impact of stillbirth on bereaved parents, highlighting the importance of clear and sensitive communication from professionals [13]. A Spanish study [14] explores parents’ experiences of stillbirth and highlights the importance of tailoring support and systems according to their specific needs. There are few studies focusing on the early experiences of parents when faced with stillbirth [15, 16]. Hence, the aim of this qualitative in-depth study was to explore parents’ initial experiences, perceptions, internal processes and coping strategies, from the awareness of reduced or absent foetal movements or other serious pregnancy complications until one month after stillbirth.

## Methods

### Design

A descriptive design was used, involving individual in-depth interviews with parents who had experienced stillbirth.

### Sample

The sample consisted of parents who had experienced a stillbirth after 22 completed weeks of gestation, interviewed approximately one month after the stillbirth.

### Data collection

Managers of all women's clinics in Sweden, and the *Swedish Infant Death Foundation,* were contacted and invited to participate by reaching out to parents who had experienced stillbirth. Ten out of 38 clinics responded positively and expressed their willingness to participate. Informants who wished to participate further confirmed their interest and signed a written consent form. This study constitutes the first part of a longitudinal study, which spans over two years for each participant and focuses on stillbirths occurring after 22 completed weeks of gestation.

Ten individual in-depth interviews were conducted via mobile phone between November 2021 and July 2023, approximately one month after the stillbirth. The informants included six women and four men, ranging in age from 22 to 53 years, with a median age of 31.5 years. Their marital status was either cohabiting with a partner (*n* = 6) or married (*n* = 4). The pregnancies ranged from 22 to 39 completed weeks of gestational (Mean = 34 weeks), and four of the informants had undergone IVF (in vitro fertilisation). Three of the informants had previous children.

The informants rated their current mental health using four options: ‘very good’, ‘good’, ‘partly bad’, and ‘bad’. Most of the informants were employed, and one was an adult student. The informants resided in different locations; one in a town and nine in the countryside. They were distributed across various parts of Sweden, including southern Sweden (*n* = 3), mid-Sweden (*n* = 5), and northern Sweden (*n* = 2).

Each in-depth interview ranged from 38 to 183 min (Mean = 121.1 min), for a total of approximately 20.11 h. All interviews were audio-recorded with the informants’ permission. Prior to the start of each interview, the interviewer verified that the informant was alone in a quiet, disturbance-free room. The interviews followed a semi-structured guide that began with the question, ‘Please, would you tell me what you experienced and perceived when you became aware of reduced or absent foetal movements in pregnancy *or* when you were told and became aware of the intrauterine foetal death?’ Thereafter, sequential questions focused on the different parts of the journey, such as the initial suspicion of something being wrong, receiving the diagnosis, giving birth, being discharged and the period within one month, see Supplementary semi-structured guiding manual – *one month after the stillbirth*. The guide was not strictly followed; rather, it was adapted to the parents’ narratives as they reflected on their experiences both past and present as they shared their stories.

The interviews were transcribed verbatim prior to data analysis. Recruitment efforts continued until no further participants agreed to participate in the study, resulting in a total of ten in-depth interviews. Data saturation was achieved after nine in-depth interviews, as no new information emerged.

This study was approved by the Regional Ethics Committee (Dnr: 2021–06536-01), and ethical considerations were upheld throughout the study. Each informant received both oral and written information about the longitudinal study and signed an informed consent form. Participation in the study was voluntary, and informants had the opportunity to ask questions before and after their in-depth interviews. Additionally, if any thoughts or concerns arose following the interview and the informants wished to seek professional support, they were offered the option to contact the researcher responsible for the study for advice and referral to the appropriate agency.

### Analysis

In this study, a thematic network analysis was conducted to systematise, organise, and describe the findings. Interpretation was employed during the analysis to elicit abstract themes [17]. Each author then independently analysed the data material inductively, according to Attride-Stirling’s thematic network analysis model [17], which categorises themes into three levels: basic, organising, and global [17]. Both authors individually read the transcribed data several times to gain an overall understanding of the content. Meaningful text segments, such as sentences, were then organised into the aforementioned three levels: basic themes were merged to form organising themes, and organising themes were grouped together into a global theme. Lastly, patterns were interpreted and clarified with verbatim quotes. The interview number, gender, number of parity or pregnancy, and age of the informants are presented below in the Results section. The authors discussed and revised all analytical decisions until consensual validation was achieved. See Table 1 for the analytical scheme.

**Table 1:** Example sentences, basic themes, organising themes and global theme

Sentence	Basic theme	Organising theme	Global theme
It was different in my womb on Tuesday so I called my midwife who said call the hospital	Awareness of changes in pregnancy and parental involvement		
		Following the journey – from suspicion to acceptance	
It was horrible to carry a dead baby inside the body, and it was scary to give birth	Caring, and giving birth and spending time with the stillborn baby		
			Stillbirth is a roller coaster, from the bright miracle of life to the deepest black grief and bottomless pain
Almost all of the informants experienced the treatment and support as human and trusting and very good at hospital	Professional treatment and support during stillbirth and postpartum		
		Support and structured activities and processes after stillbirth	
We wish a future pregnancy with a baby growing up with us	Parents’ hope for a new pregnancy		

## Results

**Fig. 1:**
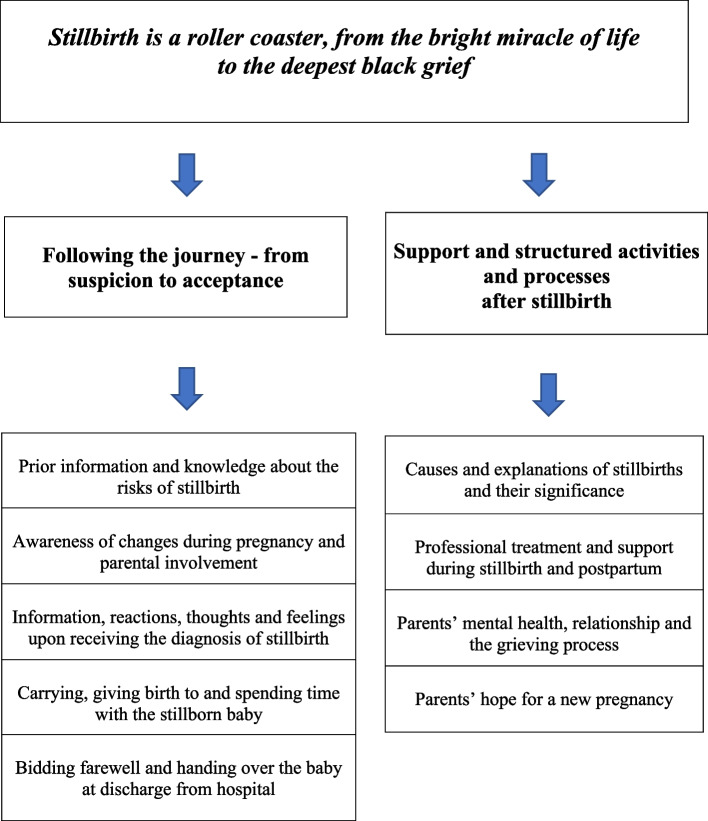
Network of global, organising and basic themes describing parents’ experiences and perceptions of stillbirth during pregnancy, birth and postpartum and until one month after stillbirth

The informants generously shared their lived experiences, internal feelings, and deep grief following the stillbirth. The in-depth interviews assumed a natural flow, and the informants displayed significant interest in sharing their traumatic, severe, and irreversible life-changing experiences. From the data, a thematic network was developed, and nine basic themes emerged, from which two organising themes were created: ‘*Following the journey – from suspicion to acceptance’* and *‘Support, structured activities and processes after stillbirth’*. Together, these organising themes constituted one global theme: *‘Stillbirth is a roller coaster, from the bright miracle of life to the deepest, black grief and bottomless pain’*. See Fig. 1.

The global theme encapsulated the informants’ perceptions and experiences, from the initial suspicion or confirmation of stillbirth, through giving birth, to the final moments of handing over their baby at discharge, and up to one month after the stillbirth. This theme also highlights the importance of professional support, clear information, and the partner relationship in helping parents cope with their grief.

Nearly all informants emphasised the priceless value of holding their stillborn baby on their chest or in their arms after birth, especially during the first moments when the baby was still soft and warm. Additionally, almost all parents cherished the time spent together with their stillborn baby postpartum, during which they tenderly cared for and welcomed the stillborn baby as a new member of their family. They highlighted the importance of maintaining close physical proximity to their baby as much as possible, recognising that their time together was limited and viewing this closeness as the most meaningful way to comprehend and cope with the tragic situation. Parents also preserved valuable and poignant mementos, such as photographs and hand and footprints of their stillborn baby, to keep for the future. Being separated from their stillborn baby at discharge was described as the worst thing ever in their lives. Experiencing a stillbirth affected the partner relationship, parents’ mental health and the grieving process. However, for some, there was a small light in the tunnel in the hope of a future pregnancy.

The organising theme *‘Following the journey – from suspicion to acceptance’* comprised the following basic themes: ‘Prior information and knowledge about the risks of stillbirth’; ‘Awareness of changes during pregnancy and parental involvement’; Information, reactions, thoughts, and feelings upon receiving the diagnosis of stillbirth’; ‘Carrying, giving birth to and spending time with the stillborn baby’; and ‘Bidding farewell and handling over the baby at discharge from hospital’. The organising theme comprises the process from the initial suspicion of something being wrong in pregnancy, through the birth and postpartum period in hospital, to the farewell when the baby was discharged.

### Prior information and knowledge about the risks of stillbirth

The informants’ knowledge about the risks associated with stillbirth differed significantly. Some described that they had not received any information from antenatal midwives about reduced or absent foetal movements in pregnancy or other serious causes that could lead to a stillbirth. One informant stated: ‘The midwife referred me to 1177 (the digital national healthcare advice service), but nothing was available to read about decreased foetal movements – I think it would be better to talk about it’ *(interview no. 2, man, first pregnancy, 32 years).* A mother of two older children affirmed that her midwife had never informed her about minor or complete cessation of foetal movements, or about the possibility of a baby dying in the womb. She knew that it could happen, but only in the context of a delayed birth. Furthermore, one informant mentioned, ‘The midwife indicated only smoking and travels as risks during pregnancy’ *(interview no. 10, man, first pregnancy, 39 years).* In contrast, two other informants clarified that they were already aware of the risks associated with reduced or absent foetal movements and understood that stillbirth could occur at any point during pregnancy. The first informant had read a lot about it on her own, while the other had experienced the loss during childhood, when a younger sibling died in their mother’s womb.

### Awareness of changes in pregnancy and parental involvement

Several women described changes in their wombs during the later stages of pregnancy, such as reduced or absent foetal movements. Some informants acted immediately and sought healthcare, while others did not. The time taken from suspicion to action ranged from just a few hours to four days after registering changes in the foetal movements. One informant described: ‘It felt different in my womb on Tuesday, so I called my midwife, who told me to call the hospital – but I told her that the baby usually moves in the evening and that I wanted to wait, so we went to the hospital on Wednesday’ *(interview no. 1, woman, primipara, 27 years).* Some female informants tried to stimulate foetal movement by pushing on their abdomen, drinking cold water, and then waiting for some movements, without any result. Conversely, some women were unaware or did not register any decrease in foetal movements at all and received the unexpected diagnosis of stillbirth when they arrived at the hospital. Their reasons for seeking healthcare included experiencing labour pains and bleeding, prelabour rupture of membranes (PROM), and undergoing a routine check-up for the baby’s growth. One woman stated, ‘I went to the hospital directly with labour pains and bleeding, and I didn’t know then that the baby was already dead’ *(interview no. 3, woman, 1-para, 31 years).*

### Information, reactions, thoughts, and feelings upon receiving the diagnosis of stillbirth

All informants received calm and matter-of-fact information from obstetricians and midwives at the hospital, who informed them that their baby had died in the womb. The parents expressed various reactions to this information, from shock to disbelief. For some parents, the emotional reaction came directly, while for others, it was not until after discharge.

Several parents found it difficult to believe the information received. Others reacted with visible expressions of shock, such as crying and shaking, while some remained silent, and showed their understanding through glances and body language. One informant said: ‘We were very sad; it was completely unimaginable. It was just over, and there was nothing to be done – the pregnancy was over’ *(interview no. 6, woman, primipara, 39 years).* Another informant described this unreal situation, where the midwife could not hear any heartbeats and then an obstetrician arrived and confirmed the absence of heartbeats – and after some minutes, another obstetrician came and confirmed the same thing. One woman described that she did not remember what she and her partner had said to each other during this conversation at the hospital and did she react with feelings until they got home. Some male informants described receiving the tragic news as unbearable, expressing feelings of anger and disappointment at the sudden loss of the life task of raising and caring for a baby. One of them stated: ‘It was terrible; I had never felt such intense pain in my life before’ *(interview no. 4, man, first pregnancy, 22 years).* Another man described feeling as though the whole world had collapsed, experiencing unreal feelings and a sense of not being awake.

Furthermore, he felt he had lost something deep within his soul, feeling different and uncertain if this feeling would persist. A female informant expressed the profound difficulty of losing her baby, particularly when it was towards the end of pregnancy and her previous pregnancy had gone well. Overwhelming feelings of guilt were also evident in the narratives, especially from a female informant, who blamed herself deeply and strongly and recurrently for not paying enough attention to the signs of decreased foetal movements. Some informants expressed profound sadness at witnessing their partners’ trauma and felt as though their life path had been irrevocably altered. The trauma of stillbirth led some participants to avoid contact with hospital staff.

### Carrying, giving birth to and spending time with the stillborn baby

The narratives disclosed diverse feelings about carrying a deceased baby inside the body. Some women reported horrible and unpleasant thoughts and feelings about carrying a dead baby inside them, expressing they were scared and worried about giving birth to a stillborn baby vaginally. Furthermore, some informants feared facing their stillborn baby and wondered what it might look like. One informant stated: ‘The dead baby is only a shell in my womb now, and I regret that no photos were taken of my womb during pregnancy’ *(interview no. 5, woman, 1-para, 31 years).* Others did not find this particular situation frightening per se, but they thought more about the fact that they would soon be separated and having to say farewell to their baby. In addition, losing the strong maternal-foetal attachment made women feel sad, as if they were losing a dear friend.

The birth of the baby evoked a variety of feelings. Parents reported overwhelming feelings of love and pride, imbedded in sadness and a strong bond with the baby. Nearly all informants recalled the birth experience four weeks postpartum, with clear overall memories. All except two of the births proceeded vaginally, but two informants underwent emergency caesarean sections due to complications. Despite the tragic circumstances, all of the informants were satisfied with the birth experience, and almost all felt the pain relief received during birth was adequate. After birth, they wished to keep their stillborn baby close. Several women expressed that it was important to have skin-to-skin contact with their baby directly after birth. One woman considered it important for her partner to hold the stillborn baby in his arms while the baby was still soft and warm.

One woman described holding her baby in her arms as the greatest and most beautiful thing that had ever happened to her. Another woman regretted not having her stillborn baby skin-to-skin, either at birth or afterwards. Caring for the stillborn baby in the same manner as they would a living baby was deemed important to the parents. This included wiping the baby when needed, putting on a diaper, dressing the baby, and ensuring that the baby appeared comfortable in bed.

A male informant expressed being overwhelmed with emotions when his stillborn baby was born, cherishing every moment. He described feeling an awesome feeling while holding him, 2850 g of love, experiencing a strong and proud father-and-son connection. Fathers also acknowledged that their expectations of what it would be like to face a stillborn baby changed once the baby was born. Many fathers were initially unaware of the impact of holding their baby close in their arms after birth. Holding the baby made them feel like fathers and parents forever. One male informant stated: ‘I felt sad but not uncomfortable; there was no rush for him to go out. He is our baby, even though he is not alive’ *(interview no. 7, man, first pregnancy, 53 years)*. However, there were a few informants who did not wish to have the stillborn baby on their chest or hold them in their arms for a longer time after birth. They wanted to say farewell as soon as possible.

The time parents spent with their stillborn babies after birth varied from one hour to 98 h, with an average of 44.8 h. Seven informants were informed about the option to spend additional time with their stillborn baby at home using a Cubitus baby (a cot equipped with cooling blocks to preserve the baby’s body). Despite that, all of the informants declined to take their stillborn baby home. Reasons cited included discomfort and concerns about the baby’s small and fragile state, and concerns about siblings at home. One female informant expressed her desire to keep the tragic situation within the hospital, fearing that returning the stillborn baby back to hospital after having taken the baby home would make it more difficult to cope with the situation. However, all informants agreed on the importance of routinely preserving memories of their baby for the future. Most parents kept mementos such as photos, hand and footprints, and plaster casts of their baby’s hands and feet. Almost all parents managed to take their own photos, and some even recorded videos. One informant conveyed the sentiment: ‘Everything that belongs to him has so much value for us, even though we don’t have him physically at home’ *(interview no. 2, man, first pregnancy, 32 years).*

### Bidding farewell and handing over the baby at discharge from hospital

For almost all informants, the act of leaving and handing over their stillborn baby to professionals upon discharge from the hospital was intolerable and difficult. Some informants described making the decision to hand over their stillborn baby after noticing changes in the baby’s body or out of a deep sense of respect for their baby’s dignity. It was crucial for them to entrust the stillborn baby to a trustworthy and caring individual. In most cases, the baby was handed over to a known midwife; however, in some instances, the baby was handed over directly to a pathologist. Many informants appreciated being given the opportunity to visit their stillborn baby at the pathology department or mortuary before the funeral. One female informant shared: ‘We visited him several times. It became a sanctuary; we cherished these moments, we held him and patted him – he was the most beautiful baby we had seen’ *(interview no. 6, woman, 0-para, 39 years).* Some informants visited their stillborn baby multiple times before the funeral, even though they noticed slight changes in the baby’s appearance. They felt reassured knowing that their baby was resting comfortably in the coffin. A male informant expressed how he preferred to focus on the joy before the sorrow, while a female informant acknowledged both the grand and beautiful aspects, along with the painful and terrible ones. Some informants recalled specific interactions with their partners during this time, with the stillborn baby’s non-living status not being the focal point. However, two informants described a paradoxical experience – the stillborn baby’s hands were described as beautiful and soft, yet the sight of the cold and blue body lying alone on a bench in the labour room was unsettling.

The second organising theme *‘Support and structured activities and processes after stillbirth’* comprised four basic themes*:* ‘Causes and explanations of stillbirths and their significance’; ‘Professional treatment and support during stillbirth and postpartum’; ‘Parents’ mental health, relationship and the grieving process’; and ‘Parents’ hope for a new pregnancy’.

This theme incorporated the importance of getting answers about the cause of the stillbirth receiving professional support, coping with mental health challenges faced by the parents, their relationship and the grieving process, up to one month after birth. Finally, there was hope for a subsequent pregnancy despite the complex emotions and health challenges involved. This theme comprised four basic components.

### Causes and explanations of stillbirth and their significance

Almost all informants did not receive any explanations or information about obvious reasons behind the stillbirth. One female informant had not received any explanation and felt that it was meaningless, particularly as she opted out of an autopsy for her stillborn baby. A few informants had received partial explanations. For instance, one male informant was immediately informed of the cause of the stillbirth at the hospital, where the obstetrician showed them on an ultrasound that the stillborn baby was lying with its stomach downwards, resulting in a kinked umbilical cord. Another informant mentioned having been informed during a routine ultrasound about the baby’s heart defect and subsequent heart failure. A father had some knowledge of the cause and considered it important for future pregnancies to be able to respond if something deviated. Nearly all informants emphasised the importance of receiving the cause and explanation of stillbirths, both for their current understanding and for future pregnancies. One female informant expressed that knowing why her baby died in her womb would alleviate the constant need to question and wonder about it. The informants did not blame healthcare professionals for the stillbirths, considering them innocent in these occurrences.

### Professional treatment and support during stillbirth and postpartum

Almost all informants encountered various professionals, including midwives, obstetricians, psychologists, social workers, deacons, priests, and pathologists at the hospital. They described the treatment and support as compassionate, trusting, and significant, with professionals providing calm and supportive care throughout the process. While most informants were satisfied with the treatment, a few requested more information and guidance during labour and the postpartum period. Some highlighted the value of the postpartum time and the support received from professionals, appreciating their ability to understand and empathise with their experiences. One female informant pointed out: ‘I could feel that she was my baby and I was her mother. I stayed with her as long as possible’ *(interview no. 8, woman, 2-para, 29 years).* However, a few informants desired more information, such as pain relief options, labour sessions, postpartum care, autopsy procedures, and funeral arrangements. One informant requested one-to-one care from a midwife, feeling disturbed by having to share the midwife with other women during labour. Another informant suggested that obstetricians could demonstrate more empathy. Professional support was highly appreciated, particularly in terms of practical assistance, such as guidance in taking photos of the baby and small acts of kindness and affirmation of the baby. One woman expressed: ‘The professionals confirmed that he was so beautiful, and they were so receptive to what we wanted, and they advised us to take our own photos of the baby, which I appreciate so much now’ *(interview no. 9, woman, 0-para, 36 years)*. However, some informants expressed a need for more support and reminders from professionals to take photos and spend time holding the stillborn baby close, especially when they felt tired or confused after the birth. Parents also emphasised that they had a strong desire to engage with their personal network. Several parents mentioned that visits from extended family, relatives, and close friends at the hospital provided a sense of unity and support, alleviating feelings of loneliness and fostering a sense of togetherness around the stillborn baby, which they believed could be beneficial for them in the future.

### Parents’ mental health, relationship and the grieving process

The informants self-assessed their current mental health as ranging from very good to very bad. Six informants rated their mental health as very bad, including five women and one man, while one informant rated it as partly bad. Some of the women who rated their mental health as very bad experienced symptoms such as waking up at night with panic attacks triggered by flashbacks and difficulty breathing. One woman reported feeling very tired and still cried a lot every day, although she no longer experienced the anxiety and panic feelings she had felt in the hospital. Some women felt depressed and were unsure of how to feel better, while others managed to overcome the most difficult moments despite facing significant challenges. One female informant rated her mental health as good and expressed a positive outlook on most aspects of her life, finding it easier to think positively rather than focusing solely on difficulties. Two male informants rated their mental health as good or very good. One mentioned receiving strong support from his partner and engaging in conversations with others, while the other expressed being open-minded and capable of processing his feelings in a positive manner, which allowed him to move through sadness more quickly.

Nearly all informants described their relationships with their partners as stronger and more stable following the stillbirth. They reported an increase in love and trust, a sense of growing together, and heightened closeness during times of strain. A female informant expressed: ‘We have been through this together, and we feel that we have each other and love each other, but you can’t take anything for granted’ *(interview no. 3, woman, 1-para, 31 years).* A male informant affirmed: ‘This tragic situation has made us closer, and we are stronger together’ *(interview no. 10, man, first pregnancy, 39 years).* However, one woman felt bitterness towards her partner, feeling that she bore the brunt of the struggle while her partner avoided discussing the stillborn baby.

The grieving process involved conversations with partners, professionals, extended family, friends, and co-workers. Almost all informants experienced profound sadness and cried frequently, struggling with poor relaxation and sleep. They harboured deep and painful feelings and thoughts about the stillborn baby, seeking solace in discussions, particularly with their partners. Professional conversations were deemed important and necessary for understanding the tragic situation. Informants believed that frequent discussions about the stillborn baby were crucial for progressing through the grieving process. Some chose to openly acknowledge their parenthood and the existence of the stillborn baby to others, despite the baby not being physically present.

Memories of the stillborn baby played a crucial role in the grieving process and coping mechanisms. Parents frequently revisited these memories, especially by looking at photos of the stillborn baby several times a day, particularly during the first month after the stillbirth. Additionally, some informants described participating in both a name-giving ceremony at the hospital and a burial ceremony for the stillborn baby in church. These ceremonies were seen as ways to honour and respect the baby, making their existence visible and dignified, and were considered integral parts of the grieving process. However, one informant, did not participate in the burial ceremony due to strong religious traditions and had not visited the grave. Visiting the grave held different significance for the informants. For some, it was a crucial, tranquil, and peaceful place where they could communicate with their buried baby. Others viewed it as an important place to tend to, adorned with stuffed animals, flowers, and candles in memory of the baby. Initially, some informants visited the grave once or twice daily.

### Parents’ hope for a new pregnancy

The hope for a subsequent pregnancy was coloured by both wishes and fears. All informants expressed a desire for another pregnancy. Nearly half of them had undergone IVF pregnancies that resulted in stillbirths. Some expressed awareness of the uncertainty surrounding IVF attempts resulting in pregnancy and hoped for a healthy, living baby next time. Informants voiced fears and worries about future pregnancies but also expressed a strong desire for subsequent siblings. Both female and male informants highlighted similar wishes for future pregnancies, including several and more frequent check-ups with midwives and obstetricians, particularly during the second trimester, as well as a controlled start to labour at specific gestational weeks using induction methods. Some informants emphasised the importance of extended professional and psychological support in case of a future pregnancy.

## Discussion

The main findings of this study, described in the overarching global theme: ‘Stillbirth is a roller coaster, from the bright miracle of life to the deepest black grief and bottomless pain’, consist of themes that emerged from the individual experiences of the participants, tracing their journey from the initial awareness of bodily changes during pregnancy to the stark reality of stillbirth. The analysis highlighted the profound impact of this tragic, irreversible life event, starting from the onset of suspicion during pregnancy and extending through the concrete confirmation of stillbirth, with a continuum of emotions and experiences in the first month thereafter.

The organising theme *Following the journey—from suspicion to acceptance* showed that stillbirth, an undesirable event, is difficult to prepare for. The results unveiled that *Prior information and knowledge about the risks of stillbirth* were lacking*,* specifically about decreased foetal movements and their association with stillbirth. Consequently, parents were neither aware nor prepared for the possibility that a stillbirth could occur during pregnancy, although there was some knowledge of the diagnosis of stillbirth. In the antenatal programme in Sweden, where women usually meet the same midwife during 9–10 visits, it is recommended by the National Board of Health and Welfare that pregnant women receive information about foetal movements and the risks of stillbirth [18].

In addition, the result also demonstrated that, in the majority of cases, there were different levels of *Awareness of changes during pregnancy and parental involvement* at various stages of pregnancy, which were later confirmed at the hospital. Discrimination attributed to stillbirth and missed warning signs has been documented in prior research [11]. The failure to pay attention to reduced or absent foetal movements in the womb, and the subsequent lack of appropriate action, sometimes resulted in feelings of guilt and shame. This was interpreted as a failure to properly safeguard the pregnancy, leading to self-perceptions of being a negligent or inadequate mother. The findings of the present study align with previous studies that have highlighted similar experiences of stigma, including feelings of shame, blame, [11, 19] and a sense of devaluation of motherhood following stillbirth [20]. Parents’ reactions upon receiving the diagnosis of stillbirth were reflected in the theme *Information, reactions, thoughts, and feelings upon receiving the diagnosis of stillbirth.* Similar to other traumatic events, parents facing stillbirth might react differently, and it is well known that they may experience a variety of feelings such as anger, sadness, mistrust, shock reactions, and panic attacks, as shown in a recent meta-synthesis [20].

The birth of the baby and the immediate postpartum care were of utmost importance, as demonstrated in the theme *Carrying, giving birth to and spending time with the stillborn baby.* The results showed that carrying a deceased baby was sometimes perceived as frightening; however, it also provided a sense of safety in knowing where the baby was, regardless of its condition. The experience brought about deep fears and inconveniences for the parents, placing them in a completely new and unavoidable situation in life, with the inevitability of giving birth to the stillborn baby within a few days. This aligns with previous studies indicating that limiting the period between the diagnosis of intrauterine death and the induction of labour decreases the risk of anxiety [21]. All labours, except for two, resulted in vaginal births. This finding is consistent with a previous study, which demonstrated that vaginal birth is the most common and recommended mode of delivery in cases of stillbirth, whether the labour occurred spontaneously or was induced, regardless of whether the individual had previously undergone a caesarean section [22]. Hence, it emerged that vaginal birth became the best outcome for both physical and emotional well-being. Keeping the stillborn baby close and repeatedly holding it, especially through skin-to-skin contact, promoted the grieving process. Previous studies have supported the overall beneficial effect of holding a stillborn baby [23, 24]. The parental role emerged and became clear when closeness, openness and love were expressed towards the stillborn baby for a longer time together. This scenario harks back to the early 1980s, when the practices and procedures surrounding stillbirth were vastly different in Swedish healthcare. During that time, professionals often treated stillbirth as a non-event, neglecting to provide adequate care or support. This approach was motivated by a desire to shield parents from emotional attachment and to encourage them to forget about the stillborn baby as quickly as possible [25]. In reality, this meant that immediately after birth, the stillborn baby was separated from the parents and swiftly taken away from the labour room, never to be seen again. Under this approach, parents were rarely given the opportunity to see, hold, or bid farewell to their stillborn baby. As a result, the stillbirth remained invisible to the parents, as well as to others and society at large. However, a previous study described that treating stillbirth as a non-event has largely, if not entirely, been abandoned in Sweden [26]. Furthermore, parents deprived of the chance to physically and emotionally care for and mourn their stillborn babies likely harboured unprocessed and deeply sorrowful memories, thoughts, and fantasies about their loss for years to come.

*Bidding farewell and handing over the baby at discharge from hospital*

Leaving the baby at the hospital upon discharge was described as the most painful experience to the majority of parents. It was important that this phase was conducted in a respectful way, and parents acknowledged the professionals who treated the stillborn baby with dignity. This finding is similar to those reported in a meta-synthesis of parents’ experiences of stillbirth, which showed that personification and a respectful attitude were of utmost importance [20].

The organising theme *Support and structured activities and processes at stillbirth* not only described the impact of receiving an explanation for the stillbirth but also highlighted the importance of professional treatment. Moreover, this theme covered the effects of stillbirth on parents’ mental health, relationships and the grieving processes, and finally, their hopes for the future.

First, the basic theme *Causes and explanations of stillbirth and their significance* showed that parents were mostly unaware of the cause and explanation behind the stillbirth, which was unsatisfactory, particularly in relation to future pregnancies. A previous Swedish study underscored the importance of understanding the cause of stillbirth [4].

The results also revealed generally satisfying experiences with the treatment and communication from professionals following stillbirth. This support was described as credible, respectful, knowledgeable, and responsive, reflecting the basic theme *Professional treatment and support during stillbirth and postpartum.* However, parents expressed a desire for more experiential and fact-based information about labour and the postpartum period. This theme described the postpartum period as friendly, humane, and supportive. The professional support encouraged parents to share their experiences and to find a balance between seeing the beauty and the sadness of their situation simultaneously. This approach facilitated that every stillborn baby was unique and valuable, deserving of unlimited time for understanding, reflection, and respect, which ultimately aided in the grieving process. Similarly, a recent Swedish systematic review identified four key aspects of care and support following stillbirth: personification, a respectful attitude, existential issues, and stigmatisation [20], which highlights the complex impacts that stillbirth had on everyone involved. Additionally, the presence of extended family and close friends at the hospital, as well as at the funeral, was important for sharing the sadness of this irreversible, life-changing event, both in the moment and for the future. However, it is worth noting that not all mothers attended the funeral due to their religious traditions, which may have hindered or delayed their ongoing grieving process.

The basic theme *Parents’ mental health, relationship and the grieving process* showed different strategies for coping with the grief, ranging from panic attacks and severe depression to feelings of exhaustion and excessive sleeping. Some parents also attempted to adopt a more positive outlook on their grief, choosing not to solely focus on the problems. However, problems arose when mental health was rated as very poor, particularly when values rooted in religious traditions precluded discussions about the stillborn baby, both with partners and relatives. This approach may have created an unhealthy bubble of loneliness that hindered the grieving process. A previous systematic review has confirmed poorer mental health outcomes following stillbirth, which is consistent with the present findings [27]. Furthermore, there were limited treatment options available for those who reported worsening mental health following stillbirth, highlighting the need for further research in this area [28]. Moreover, this theme included the partner relationship, showing that the bond between partners, in most cases, was usually strengthened after the stillbirth. Crying, talking, and reminiscing together, often repeatedly, became necessary and healing aspects of the grieving process. Seeking support and engaging in discussions with professionals and others in their network were also very important for coping with grief. Moreover, the theme included memories of the stillborn baby, which served as the only tangible connection that could be retained in life and were considered incredibly important for navigating the grieving process, often revisited multiple times daily. A previous study confirmed that when a baby is stillborn, staff on labour wards usually encourage parents and other family members to take photographs and gather other mementos [29]. Visiting the baby’s grave became an important part of the mourning process, allowing to feel closer to their buried baby.

Grief is considered a normal reaction after stillbirth, but the psychosocial consequences of stillbirth can last for a long time and may increase the risk of mental illness [28]. It is therefore important to develop supportive treatment options that extend beyond the immediate period after stillbirth.

Finally, the result showed that it was common for parents to desire a new, healthy pregnancy in the future, which was reflected in the basic theme *Parents’ hope for a new pregnancy*. Nearly half of the informants reported that their IVF pregnancies ended in stillbirth, a relatively common occurrence, which is consistent with a systematic review indicating an increased risk of stillbirth associated with IVF pregnancies compared to natural conception [30]. In the case of a future pregnancy, the importance of receiving continued high-quality healthcare, along with more information about pregnancy risks, examinations, assessments, and preventive measures, was highlighted. Some studies have investigated the provision of care for parents during pregnancies following stillbirth. A UK study [31] provided numerous examples of good practice. However, that study indicated the overall lack of consistency in delivering adequate emotional and psychological support for women during pregnancies after stillbirth. Another study underscored the importance of tailoring support systems not only to mothers but also to fathers, who often reported a lack of opportunities to grieve [19]. A third study emphasised the need for greater attention to providing thoughtful, empathic, and collaborative care in all pregnancies following stillbirth [32].

### Methodological considerations

The study has some limitations. Its qualitative nature preluded any quantitative claims about the individuals affected by stillbirth. Only ten informants were included in the study, and no new data emerged after nine interviews. Since stillbirth is a rare phenomenon, recruiting parents for longitudinal studies can be challenging. However, it is possible that including more participants might yield new insights and nuances in the results. Using mobile phones in the present interview study can be seen as beneficial, as the subject area itself contains very private and sensitive thoughts, feelings and experiences, where physical presence may feel more stressful. A negative aspect of using mobile phones is the inability to recognise the body language of the informants, which could lead the interviewer to miss expressions of discomfort that need to be addressed.

The authors’ pre-understanding and experiences of this topic may have influenced both the research process and the results. Both authors are midwives, and when discussing the analysis, they focused on their prior understandings [17]. As midwives typically interact with individuals who are in the process of starting a family, including childbirth with a living baby, they have rarely engaged with individuals affected by stillbirth, resulting in a distinctly outsider perspective.

It should be noted that all informants represented a crystallised group of individuals who were affected by an irreversible, tragic, live-changing event who wanted to participate in the study to share their experiences, perceptions, values and deep sadness. The feelings of loss and deep sorrow reported by the affected individuals were based on their individual and personal mental states one month after the stillbirth. A longitudinal study is ongoing to explore the experiences of these individuals over time following this irreversible and tragic life-changing event. Trustworthiness is an important aspect of qualitative research, which includes evaluating the similarities and differences between themes [33].

Dependability is one way to ensure trustworthiness. In addition, trustworthiness was enhanced by describing the process in detail to allow the reader to follow the analytical process. The authors, being experienced midwives familiar with qualitative methods, further ensured the trustworthiness of the translation. Special attention was paid to the citations to maintain the integrity of the data. Performing the steps in the analysis process both individually and jointly ensured credibility and confirmability. Transferability was assured by providing detailed descriptions of the content and context of the interviews, as well as the selection and characteristics of the participants, data collection, and the analysis process and findings.

One strength of the study was that the informants were recruited from a wide geographical region that included both sparsely and densely populated areas. Furthermore, we had information about the informants’ socio-demographic and ethnicity backgrounds. The informants were followed from the point of awareness and suspicion of reduced or absent foetal movements at home, or when seeking healthcare for other important reasons during pregnancy, until one month after the stillbirth. Due to the different scenarios surrounding each stillbirth, the opening question in the in-depth interview was asked in two different ways. The first author conducted all the in-depth interviews according to the same interview guide, ensuring that all protocols for conducting in-depth interviews and analysing data were carefully followed. The basic themes were corroborated by quotes from the informants. Finally, both study authors collaborated in collecting and analysing the data, as well as interpreting the results.

## Conclusion

This study sheds light on the profound and devastating impact of stillbirth on parents, who are confronted with the loss of their long-awaited and cherished baby without an immediate understanding of why it occurred. The intense grief and pain experienced by parents in the first month after stillbirth is described as a heavy burden that persists day and night, reflected in their poor/very poor mental health. Despite the immense challenges faced by parents, the study highlights the importance of developing individual strategies to cope with this tragic and irreversible life-changing event.

## Supplementary Information

Supplementary Material 1.

## Data Availability

Our data (individual in-depth interviews) cannot be shared openly due to participants privacy concerns.
