# Supplementary material for: Is it possible for parents to endure a stillbirth? Initial experiences, perceptions and strategies: individual in-depth interviews in Sweden 2021–2023
Source: BMC Pregnancy Childbirth. 2025 Jan 3;25:4. doi: 10.1186/s12884-024-07055-0 (PMC11699641; doi:10.1186/s12884-024-07055-0)
Supplement: Supplementary file 1 — Supplementary Material 1. [file 12884_2024_7055_MOESM1_ESM.docx]

**Supplementary semi-structured** **guiding manual – *one month after the stillbirth***

The initial question: ‘Please, would you tell me what you experienced and perceived when you became aware of reduced or absent foetal movements in pregnancy *or* when you were told and became aware of the intrauterine foetal death? Thereafter overall sequential questions:

1. Would you tell me about your information/knowledge about the risks of a stillbirth during the pregnancy?
2. How did you become aware of the pregnancy complications?
3. How did you act when you became aware of the pregnancy complications?
4. When you received the diagnosis of the stillbirth at hospital – what kind of information did you receive initially, and what were your first thoughts/feelings/reactions upon this tragical and irreversible moment?
5. Would you tell me about carrying/giving birth to/spending time with the stillborn baby at hospital?
6. How would you describe your thoughts and feelings about bidding farewell/handing over the stillborn baby at discharge from hospital?
7. Do you know the cause/s of the stillbirth?
8. Have you got an explanation/s why the stillbirth occurred?
9. Is it significant to know why the stillbirth occurred? Please, explain why you think it is significant or not significant to know why the stillbirth occurred!
10. Would you tell me how you experienced the professional treatment and support at hospital -during stillbirth/the postpartum?
11. How would you describe/assess/request during the postpartum time?
12. How do you self-assess your mental health at this stage?
13. How would you describe your relationship to your partner after the stillbirth at this stage?
14. How would you describe your grieving process at this stage?
15. How would you describe your thoughts/feelings/fears/wishes for a potentially future pregnancy?
